# Navigating diagnostic dilemmas toward precision therapy: a case report and literature review on gastric metastasis from breast cancer

**DOI:** 10.3389/fonc.2026.1760937

**Published:** 2026-04-16

**Authors:** Xiaoyu Zhang, Xiangwu Lin, Dunya Yang, Xianglan Lin, Yan Zhang, Shiyu Liu, Xi Chen, Naying Yu, Qunxiang Chen

**Affiliations:** 1 Department of Oncology, 900th Hospital of PLA Joint Logistic Support Force, Fuzhou, Fujian, China; 2 Department of Oncology, Fuzong Clinical Medical College of Fujian Medical University, Fuzhou, Fujian, China

**Keywords:** breast cancer, diagnostic algorithm, gastric metastasis, immunohistochemistry, phenotypic evolution

## Abstract

Gastric metastasis from breast cancer (GMBC) is a rare but diagnostically challenging condition, whose clinical and imaging features often mimic primary gastric cancer or treatment-related adverse effects. This study integrates a detailed case of a 54-year-old woman with Luminal B invasive lobular carcinoma who developed gastric metastasis during systemic therapy, with a systematic review of 35 recent cases (2019–2024) to delineate the clinical profile and management of GMBC. In the reported case, immunohistochemical analysis revealed phenotypic evolution, with hormone receptor expression shifting from ER 80%/PR 5% in the primary tumor to ER 30%/PR negative in the gastric metastasis. Literature synthesis identified invasive lobular carcinoma as the predominant histology (57.14%), abdominal pain as the most common symptom (54.29%), and highlighted the diagnostic utility of immunohistochemical markers—particularly GATA3 (positive in 71.43% of tested cases). Treatment remains primarily systemic, with endocrine therapy demonstrating survival benefit in hormone receptor-positive disease. We emphasize the need for heightened clinical suspicion in breast cancer patients with upper gastrointestinal symptoms and propose a structured diagnostic pathway centered on endoscopic deep biopsy and comprehensive immunohistochemical profiling. Re-biopsy to assess phenotypic evolution is crucial for guiding personalized therapy, while surgical intervention should be reserved for palliation of complications or selected cases of oligometastatic disease.

## 1 Introduction

Breast cancer is a leading cause of cancer-related morbidity and mortality in women worldwide. While it commonly metastasizes to bone, lung, liver, and brain, gastrointestinal involvement is uncommon, with gastric metastasis occurring in approximately 0.04%-0.3% of clinical cases (1, 2). However, autopsy studies suggest a much higher incidence (4-35%) (3), indicating that GMBC is significantly underdiagnosed ante-mortem.

The clinical dilemma is twofold. First, symptoms of GMBC—such as epigastric pain, bloating, and nausea—are non-specific and can be easily attributed to adverse effects of chemotherapy or targeted agents (4). Second, both radiological and endoscopic appearances of GMBC can be indistinguishable from those of primary gastric cancer, especially Borrmann type III/IV or linitis plastica (5). This frequently leads to diagnostic delay or misdiagnosis, which can adversely impact patient outcomes. The average time from primary breast cancer diagnosis to the detection of gastric metastasis is 5–8 years, and most patients have concurrent metastases at other sites at the time of gastric diagnosis (6). Invasive lobular carcinoma (ILC) has the tendency to metastasize to the stomach more often compared to invasive breast carcinoma of no special type (7).

Here, we report a case of GMBC in a patient with Luminal B breast cancer, highlighting the diagnostic challenges encountered and the pivotal role of immunohistochemistry in reaching the correct diagnosis. More importantly, we observed a notable change in the hormone receptor status of the gastric metastasis compared to the primary tumor. Through a contemporary literature review and analysis of this case, we seek to move beyond mere description and provide a clinically actionable framework for the diagnosis and management of this elusive condition, with a special focus on the implications of tumor phenotype evolution.

## 2 Case presentation

A 54-year-old female presented to our hospital in August 2022 with a history of progressively worsening pain and restricted movement in the left shoulder and elbow. Previous surgery for a presumed adhesive capsulitis of the shoulder (Feb 2022) had provided no relief.

**Figure 1:**
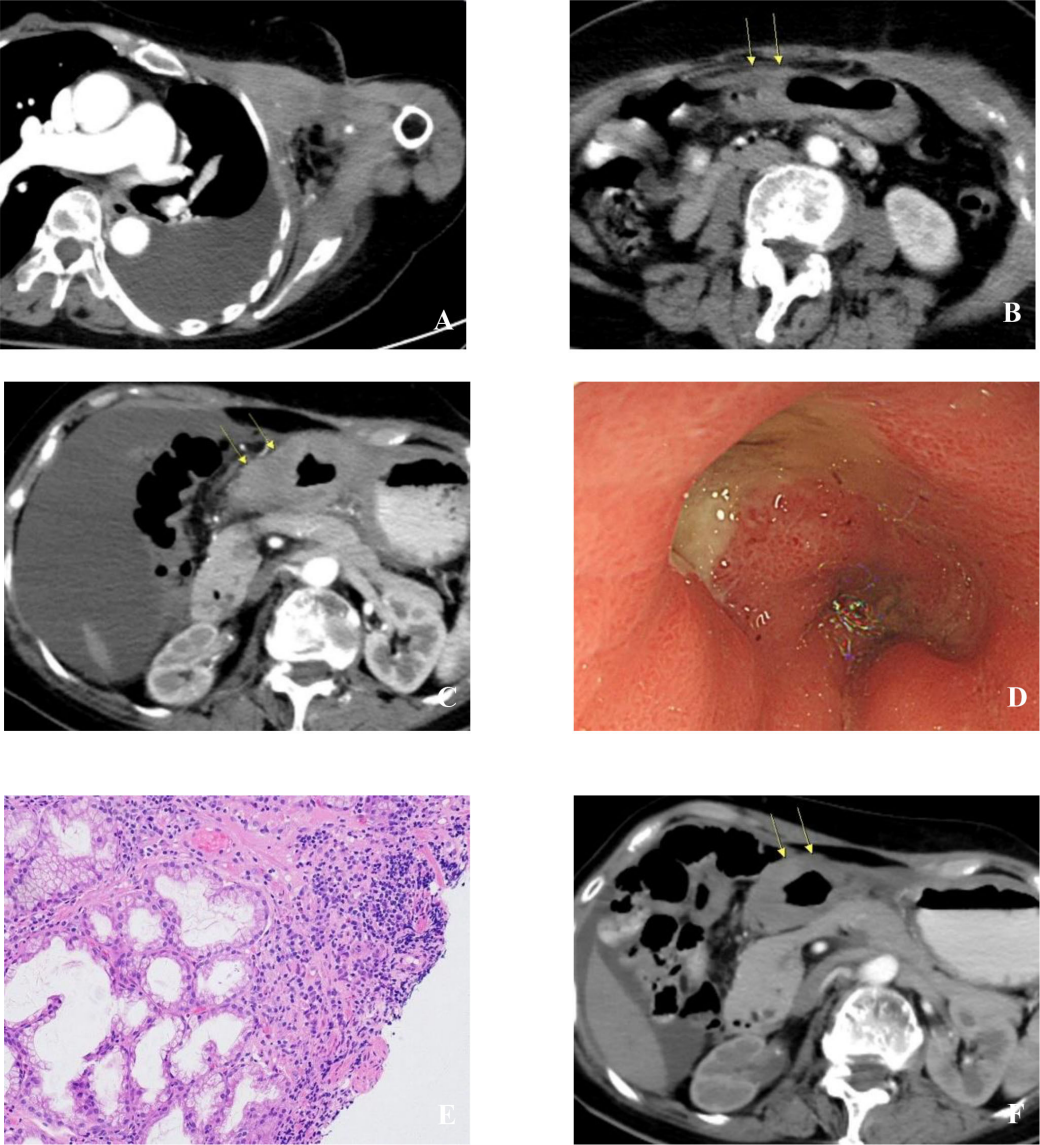
Imaging and pathological data of the case patient: **(A)** Chest CT (2022-08-24) showing extensive metastatic breast cancer in the left chest wall and massive left pleural effusion; **(B)** Abdominal CT (2022-08-24) demonstrating normal gastric wall thickness; **(C)** Abdominal CT (2024-07-02) revealing significant gastric wall thickening; **(D)** Gastroscopy (2024-07-03) showing multiple antral ulcers (suspected malignancy)? with pyloric deformation (Borrmann type III); **(E)** Gastric antral mucosal biopsy (2024-07-04) indicating focal ulceration with partial epithelial dysplasia, consistent with breast cancer metastasis based on immunohistochemical results; **(F)** Abdominal CT (2024-11-15) demonstrating reduced gastric wall thickening compared to previous imaging.

Physical examination revealed a shrunken, firm, and fixed left breast with induration of the surrounding chest wall skin. Movement of the left shoulder and elbow was severely restricted. CT scans demonstrated a left breast mass, infiltration of the left chest wall and axilla, a large left pleural effusion, and multiple osteolytic bone lesions, while the gastric wall thickness remained normal (**Figures 1A, B**). A left breast biopsy confirmed ILC, histological grade 3, with the following immunoprofile: ER (80%), PR (5%), HER2 (1+), Ki67 (30%), CK5 (–), Ecadherin (–), Calponin (–), P120 (cytoplasmic, +), P63 (–). The diagnosis was stage IV Luminal B (HER2-negative) breast cancer. Baseline tumor markers were elevated: carbohydrate antigen 125 (Ca125) at 168.6 U/ml and carbohydrate antigen 153 (Ca153) at 255 U/ml.

From August 2022 to February 2023, she received 7 cycles of first-line chemotherapy with Nab-paclitaxel and Capecitabine, achieving a partial response (PR). This was followed by maintenance therapy with Capecitabine, Abemaciclib, Exemestane, and Leuprorelin until April 2024.

In April 2024, despite stable solid lesions, rising tumor markers (Ca125: 151.3 U/ml, Ca153: 76.55 U/ml) and increased pleural/pelvic effusions suggested progression. The regimen was switched to Capecitabine, Abemaciclib, Fulvestrant, and Leuprorelin. By July 2024, the patient reported significant upper abdominal discomfort, anorexia, bloating, and weight loss. Tumor markers surged further (Ca125: 433.3 U/ml, Ca153: 153.8 U/ml). Repeat CT showed increased effusions and new, marked thickening of the gastric wall, presenting a “linitis plastica” pattern (**Figure 1C**). We reached a crossroads in diagnosis: is it primary gastric cancer or metastatic disease?

Gastroscopy revealed two irregular, depressed lesions with raised margins in the gastric antrum, involving the angle and lower body, causing deformation of the gastric antrum and pylorus (**Figure 1D**). The endoscopic impression was malignant ulcers (Borrmann type III). Histopathological examination of the biopsy showed focal ulceration with partial epithelial dysplasia. Critical IHC analysis confirmed breast cancer metastasis: ER (30%), PR (–), CerbB-2 (1+), Ki67 (40%), GATA3 (++), P120 (++), CKL (+++), p53 (10%), MUC5AC (+++, glandular epithelial positive), MUC-6 (++, intrinsic gland positive), CDX-2 (–), CMV (–), INSM-1 (–), E-Cad (–) (**Figure 1E**). A diagnosis of breast cancer with metastases to the pleura, chest wall, bone, peritoneum, and stomach was confirmed.

Given the suspected endocrine resistance, third-line chemotherapy with Eribulin was initiated. After one cycle, her abdominal symptoms improved markedly. A follow-up CT in December 2024 showed reduced gastric wall thickening (**Figure 1F**) and stabilized disease, though tumor markers began to rise again.

## 3 Literature review

Using the keywords “breast cancer” “gastric metastasis” and “metastases to the stomach”, we collected a total of 35 cases of breast cancer with gastric metastasis from 2019 to 2024 from databases such as PubMed and Wiley (**Table 1**) (4, 6, 8–38). Among these cases, 34 were female and 1 was male. The average age at breast cancer diagnosis was 59.76 years, and the average age at gastric metastasis diagnosis was 65.56 years. The average interval between breast cancer diagnosis and gastric metastasis was 5.60 years. In 6 cases (17.14%), breast cancer and gastric metastasis occurred simultaneously. The primary tumor was invasive lobular carcinoma in 20 cases (57.14%), invasive ductal carcinoma (IDC) in 11 cases (31.43%), and mixed-type carcinoma in 1 case (2.86%). Among the primary tumors, 21 cases (60%) were hormone receptor (HR)-positive, 4 cases (11.43%) were triple-negative, and 3 cases (5.71%) showed HER2 protein expression. Among the gastric metastases, 23 cases (65.71%) were HR-positive, 3 cases (8.57%) were triple-negative, and 3 cases (8.57%) showed HER2 protein expression. Further immunohistochemical analysis of gastric metastases revealed GATA3 positivity in 25 cases (71.43%), CK7 (Cytokeratin 7) positivity in 13 cases (37.14%), GCFDF15 (grosscystic disease fluid protein 15) positivity in 9 cases (25.71%), and Mammaglobin positivity in 5 cases (14.29%). The most common symptoms of gastric metastasis were abdominal pain or discomfort (19 cases, 54.29%), nausea or vomiting (7 cases, 20%), melena or hematochezia (7 cases, 20%), weight loss (6 cases, 17.14%), and decreased appetite (4 cases, 11.43%). In 5 cases (14.29%), patients were asymptomatic. CT findings primarily showed localized or diffuse gastric wall thickening (13 cases, 37.14%). Some patients presented with gastric outlet obstruction and proximal gastric dilation (2 cases, 5.71%), while 2 cases (5.71%) showed no significant abnormalities on CT. The entire stomach (10 cases, 28.57%), gastric body (9 cases, 25.71%), gastric antrum (7 cases, 20%), gastric fundus (5 cases, 14.29%), and cardia (5 cases, 14.29%) were commonly involved under endoscopy. The layers of the gastric wall most frequently involved were the lamina propria (11 cases, 31.43%), mucosal layer (7 cases, 20%), submucosal layer (5 cases, 14.29%), muscular layer (2 cases, 5.71%), and serosal layer (2 cases, 5.71%). In addition to gastric metastasis, almost all cases were accompanied by metastases to other organs, commonly including bone (14 cases, 40%), lymph nodes (9 cases, 25.71%), intestines (6 cases, 17.14%), peritoneum or omentum (6 cases, 17.14%), lung (6 cases, 17.14%), and liver (4 cases, 11.43%). The time from the detection of gastric metastasis to death ranged from a few weeks to 5 years.

**Table 1:** Summary of 35 cases of breast cancer with gastric metastasis (2019–2024).

Author, year	Sex	Initial diagnosis							Gastric metastasis										
		Age (years)	Side	Type	Expression of ER,PR and Her2	TNM staging	Stage	Treatment	Age (years)	Internal time	Symptoms	Positive immune-histochemical index	Negative immune-histochemical index	CT manifestation	Lesion site under endoscopy	Involved layer	Other metastatic organs	Treatment	Interval time from gastric metastasis to death
Okamoto, 2021 (8)	F	48	L	IDC	ER(+),PR(+), Her2 (–)	T3NxM1	IV	Endocrine therapy, chemotherapy	51	3 years	Melena, syncope	ER,PR,GCFDF15,GATA3, Mammaglobin, CK20	ND	Pylorus thickening	Whole stomach	ND	Spine, sternum, ileum, pleura, lung, spleen, lymph node	Laparoscopic distal gastrectomy and Billroth II anastomosis, chemotherapy	6 months
Husain, 2021 (9)	F	44	L	IDC	ER(+),PR(+), Her2(+)	T2N1M0	IIB	Neoadjuvant chemotherapy, operation, endocrine therapy	47	3 years	Nausea, vomiting, epigastralgia, weight loss	ER,GATA3,CK7	PR,Her2, GCFDF15	Pylorus-duodenum thickening, pylorus obstruction, gastric dilatation	Whole stomach	Mucosa	Omentum	Jejunostomy, chemotherapy	ND
Yamada, 2022 (10)	F	72	R	ILC	ER(+),PR (–), Her2 (–)	TxN3M0	IIIC	Operation, endocrine therapy	75	3 years	Epigastralgia, vomiting, edema, weight loss	ER,CK7	CK20	Antrum thickening, perigastric peritoneal nodules	Antrum, pylorus	Submucosa	Peritoneum	Pyloric stent implantation, endocrine therapy, chemotherapy	7+ months
Rech, 2023 (11)	F	69	ND	ND	ND	ND	ND	Operation, endocrine therapy	73	4 years	Weakness, mild abdominal pain, arthralgia, weight loss	ER,GATA3, Mammaglobin, Her2(equivocally positive)	GCFDF15	ND	Corpus	ND	Bone	Prednisone(paraneoplastic syndrome), targeted therapy (pertuzumab, trastuzumab), chemotherapy	6+ months
Tang, 2020 (12)	F	65	L	IDC	ER (–),PR (–), Her2 (–)	T2N1MO	IIB	Operation, chemotherapy, radiotherapy	67	17 months	Stomachache, lower back pain	GATA3, Mammaglobin	ER,PR,Her2	ND	angular incisure	ND	Left adrenal gland, abdominal cavity and retroperitoneal lymph nodes	Phase 3 clinical trial (atezolizumab/ placebo + paclitaxel)	ND
Cardoso, 2021 (13)	M	49	L	ND	ND	ND	ND	Operation, chemotherapy, radiotherapy	54	5 years	Diarrhea, epigastralgia, episodes of fever, loss of appetite, weight loss	ER,GATA3	ND	ND	Cardia, corpus, fundus	Mucosa	ND	Palliative care	2 months
Kwon, 2024 (14)	F	72	R	IDC	ER (–),PR (–), Her2 (–)	ypT3N3M0	ypIIIC	Neoadjuvant chemotherapy, operation, chemotherapy, radiotherapy	72	9 months	Epigastric pain and discomfort	CK7	GCFDF15, GATA3	ND	Fundus	Lamina propria	Para-aortic lymph nodes, bone	Laparoscopic total gastrectomy, chemotherapy, hip replacement surgery	7 months
Noor, 2020 (15)	F	34	ND	ND	ER(+),PR(+), Her2 (–)	ND	NA	Chemotherapy, radiotherapy, ndocrine therapy	68	32 years	Epigastralgia	ER,PR,GATA3	Her2	ND	Corpus	Lamina propria	Bone,sigmoid colon	Chemotherapy	2 years
Botto, 2023 (16)	F	59	R	ND	ER(+),PR (–), Her2 (–)	T1cN2 M0	IIIA	Chemotherapy, radiotherapy, endocrine therapy	61	2 years	No symptoms	GATA3,CK7	ER,PR,Her2, CK20	No abnormality	Whole stomach	Lamina propria	Bone, ascending colon	Endocrine therapy, targeted therapy (CDK4/6 inhibitor)	6 months
Fousekis, 2022 (17)	F	61	ND	ILC	ER(+),PR(+), Her2 (–)	TxNxM1	IV	Endocrine therapy, targeted therapy (CDK4/6 inhibitor)	64	3 years	Dysphagia, dyspepsia	ER,PR,CK7	Ecadherin	No abnormality	Distal esophagus, cardia, corpus, antrum, duodenum	ND	Bone,lung	Implantation of jejunal nutrition tube, chemotherapy	ND
Ito, 2023 (18)	F	77	R	ILC	ER(+),PR(+), Her2 (–)	T2NxM1	IV	Endocrine therapy, targeted therapy (CDK4/6 inhibitor)	77	0	ND	GCFDF15, GATA3	ND	ND	Whole stomach	ND	Thoracolumbar spine	Endocrine therapy, targeted therapy (CDK4/6 inhibitor)	5+ years
Ito, 2023 (18)	F	53	L	ILC	ER(+),PR(+), Her2 (–)	ND	ND	Operation, radiotherapy, endocrine therapy, targeted therapy (trastuzumab)	63	10 years	Pharyngeal obstruction	GATA3	Ecadherin	Gastric wall thickening	Whole stomach	Lamina propria	Peritoneum	Endocrine therapy, targeted therapy (CDK4/6 inhibitor)	ND
Faraz, 2024 (19)	F	72	ND	ND	ER(+),PR(+), Her2 (–)	T0NxM1	IV	Radiotherapy, endocrine therapy, targeted therapy (CDK4/6 inhibitor)	ND	5 years	Loss of appetite, abdominal discomfort, abdominal distention after eating, recurrent diarrhea	ER,PR,GATA3	Her2	ND	Corpus, antrum, pylorus	Lamina propria	Retroperitoneal lymph nodes, bone, spinal canal, duodenum, ascending colon	Chemotherapy, targeted therapy (Alpisilib)	6 months
Bai, 2023 (20)	F	30	R	IDC	ER (–),PR (–), Her2 (–)	pT3N0 MO	IIB	Chemotherapy	36	6 years	Vomiting, loss of appetite, hematemesis, hematochezia	Her2,GATA3	ND	ND	Whole stomach	Lamina propria	Lymph nodes, chest wall, pleura, left breast, thyroid lobe	Chemotherapy, targeted therapy(trastuzumab,apatinib)	14 months
Kaneko, 2020 (4)	F	45	R	ILC	ER(+),Her2 (–)	T4bN2 M0	IIIB	Neoadjuvant chemotherapy, operation, radiotherapy, endocrine therapy	52	7 years	No symptoms	ER,PR,GCFDF15,Mammaglobin	Ecadherin	Gastric wall thickening	Corpus	Mucosa	ND	Endocrine therapy, targeted therapy (CDK4/6 inhibitor)	ND
Tanaka, 2023 (21)	F	74	L	ILC	ND	T2N0M0	IIA	Operation, endocrine therapy	79	5 years	Epigastric discomfort	GCFDF15, GATA3	ER,PR,CK20	ND	Corpus	Submucosa	Axillary lymph nodes,left breast, left chest	Chemotherapy	20 months
Katuwal, 2023 (22)	F	85	R	ILC	ER(+),PR (–), Her2 (–)	T3N1M1	IV	Endocrine therapy	85	0	Occasional nausea and abdominal cramps	ER,GATA3,CK7	PR,Her2, Ecadherin	Fundus thickening	Fundus	ND	Colon	Endocrine therapy, targeted therapy (CDK4/6 inhibitor)	2+ years
Sun, 2023 (23)	F	58	R	ILC	ER(+),PR (–), Her2 (–)	cT2N0 M1	IV	Endocrine therapy, targeted therapy (CDK4/6 inhibitor)	58	0	Abdominal distention, nausea, vomiting	ER,GATA3,CK7	PR,CK20	Antrum thickening, with visible peripheral lymph nodes	Antrum-duodenum	ND	Bone	Gastrojejunostomy and colostomy, endocrine therapy, targeted therapy (CDK4/6 inhibitor)	ND
Li, 2022 (24)	F	58	R	ILC, IDC	ND	pT1cN1M0	IIA	Operation, chemotherapy, radiotherapy, endocrine therapy	61	3 years	Gastric discomfort	ND	ND	Enhancement of the mucosal and muscular layer of antrum	ND	ND	Peritoneum, bilateral adnexa	Exploratory operation, intraperitoneal perfusion chemotherapy	ND
Zhang, 2021 (25)	F	46	B	ILC	ER(+),PR(+), Her2 (–)	ND	ND	ND	46	0	Epigastric discomfort	ER,PR,GATA3, CK7	ND	Antrum thickening	Whole stomach	Epithelium	Left axillary lymph nodes, bone	ND	ND
Huang, 2023 (26)	F	61	L	IDC	ER(+),PR(+), Her2 (–)	ND	ND	Operation, radiotherapy, endocrine therapy	71	10 years	Hematochezia, acid regurgitation	ER	PR,Her2	Distal gastric wall thickening	distal stomach	Mucosa, submucosa	Lung,bone	Endocrine therapy, targeted therapy (CDK4/6 inhibitor)	ND
Liang, 2023 (27)	F	70	L	ILC	ND	pT2N3 M0	IIIC	Operation, radiotherapy	72	2 years	Vomiting, right abdominal pain, weight loss	ND	ER,PR,Her2	ND	Antrum	Lamina propria	ND	None	ND
Kimchy, 2022 (28)	F	77	ND	IDC	Her2(+)	ND	IV	Antibody-Drug Conjugate	77	1 months	Abdominal pain, melena	Her2,GATA3, Ecadherin	ND	ND	Cardia	Lamina propria	Brain,liver, lung	ND	ND
Kimchy, 2022 (28)	F	ND	ND	IDC	ER (–),PR (–), Her2(+)	ND	ND	ND	77	ND	Abdominal pain, melena	Her2,GATA3, Ecadherin	ND	ND	Cardia	Lamina propria	Brain,liver, lung	Antibody-Drug Conjugate (Trastuzumab Deruxtecan)	ND
Johnson, 2021 (29)	F	44	ND	IDC	ND	T1cN2 M0	IIIA	Operation, chemotherapy, radiotherapy	50	6 years	No symptoms	ER,GCFDF15, CK7,CK20	ND	ND	Whole stomach	Submucosa,muscular layer, serosa	ND	ND	ND
Mohy-ud-din,2019 (30)	F	73	R	ILC	ER(+),PR(+), Her2 (–)	ND	IA	Operation, endocrine therapy	83	10 years	Nausea, vomiting	ND	ND	Anrum mass-like thickening, gastric outlet obstruction, dilation of the proximal stomach	Antrum	Deep mucosa, submucosa, muscularis	ND	ND	ND
Jabi, 2021 (31)	F	60	R	ILC	ND	T1NxM1	IV	ND	60	0	Epigastralgia, hematemesis	ER,PR,GATA3	ND	ND	ND	ND	ND	ND	ND
Sasaki, 2024 (32)	F	35	ND	ILC	ER(+),PR(+), Her2 (–)	pT2N1aM0	IIB	Operation, endocrine therapy	50	15 years	Abdominal pain	ER,PR,GCFDF15,GATA3,CK7	Her2,CK20, Ecadherin	ND	Whole stomach	ND	Colon	Endocrine therapy, targeted therapy (CDK4/6 inhibitor)	ND
Kartotaroeno,2024 (33)	F	62	ND	ILC	ER(+),PR(+), Her2 (–)	ypT3N2aM0	ypIIIA	Neoadjuvant chemotherapy, operation, chemotherapy	66	4 years	Epigastric discomfort, progressively aggravated constipation	ER,GATA3	PR,Her2	Diffuse thickening of the gastric wall	ND	ND	Colon, abdominal cavity, vertebral body, lacrimal gland.	ND	2 years
Hanafiah, 2021 (34)	F	49	L	ILC	ND	ND	ND	Operation, chemotherapy, radiotherapy	71	22 years	Hoarseness, weight loss, early satiety	ER,PR,CK7	CK20	ND	ND	ND	Supraclavicular and mediastinal lymph nodes, liver, bone	Palliative care	2 years
Xu, 2024 (6)	F	71	L	ILC	ER(+),PR(+), Her2 (–)	T1bNx M1	IV	Endocrine therapy	71	0	No symptoms	ER,PR,GCFDF15,GATA3	CK20, Ecadherin	Thickening of the gastric wall with mild to moderate enhancement	Antrum	Lamina propria	Skin	Endocrine therapy	22 months
Watanabe, 2021 (35)	F	55	R	IDC	ER(+),PR(+), Her2 (–)	pT1cN1M0	IIA	Operation, chemotherapy, endocrine therapy	71	14 years	No symptoms	ER,PR,GATA3	Her2	ND	Cardia, fundus	Mucosa	Lung, mediastinum, supraclavicular region, liver, thyroid	Endocrine therapy, targeted therapy (CDK4/6 inhibitor, mTOR inhibitor), chemotherapy	33+ months
Jinushi, 2023 (36)	F	74	ND	ILC	ND	ND	IV	ND	80	6 years	Anorexia	ER,PR,GCFDF15,GATA3, Mammaglobin, CK7,Eecadherin	Her2, CK20	ND	Whole stomach	Lamina propria	Axillary lymph nodes, bone	ND	ND
Shin, 2023 (37)	F	67	R/L	ILC/ ILC	ER(+),PR(+), Her2 (–)/ ER(+),PR (–), Her2 (–)	pT3N3 M0/ pT1cN1aM0	IIIC/IIA	Operation, chemotherapy, radiotherapy, endocrine therapy/ND	72	5 years	Abdominal distention, chronic constipation, intermittent hematochezia	ER,GCFDF15, GATA3,CK7	PR,Her2, Mammaglobin, CK20	ND	Corpus	ND	ND	Endocrine therapy, targeted therapy (PIK3CA inhibitor)	ND
Sohail, 2021 (38)	F	63	L	ILC	ER(+),PR(+), Her2 (–)	ND	IIB	Neoadjuvant chemotherapy, radiotherapy, operation	69	3 years	Abdominal pain, melena, fatigue, early satiety	ER,PR,GATA3	Her2	Moderate to severe thickening of the gastric wall	Fundus, corpus	Mucosa, serosa	Peritoneum	Palliative care	Several weeks

ND, not described.

Key findings with clinical implications: ①Symptoms: Abdominal pain/discomfort (54.29%) was the most common symptom, but 14.29% of patients were asymptomatic. ②Imaging: CT most frequently showed gastric wall thickening (37.14%), but was normal in 5.71% of cases, underscoring its limited sensitivity. ③IHC profile: GATA3 was the most valuable positive marker (71.43% of tested cases), while loss of Ecadherin was a common finding in ILC metastases. A combination of immunohistochemical markers (e.g., ER+/GATA3+/GCFDF15+/CK7+/CK20-) was highly suggestive of breast origin. ④Therapeutic response: Endocrine therapy, often combined with CDK4/6 inhibitors, was a cornerstone of treatment for HR-positive cases and was associated with prolonged survival in several reports. ⑤Clinical course: The time from GMBC diagnosis to death varied widely, from weeks to over 5 years, highlighting the heterogeneous biology of the disease and the impact of effective systemic therapy.

## 4 Discussion

This case and our literature synthesis illuminate the complex landscape of GMBC. We identify three critical areas that shape patient outcomes: diagnostic challenges, tumor evolution, and therapeutic decision-making.

### 4.1 Navigating the diagnostic labyrinth: a call for a structured approach

In our patient’s case, the suspicion of a second primary malignancy are emblematic of the diagnostic odyssey in GMBC. The symptoms are non-specific, and CT findings, while suggestive, are not definitive. The endoscopic appearance can be deceptive, often mimicking advanced primary gastric cancer (39).

**Figure 2:**
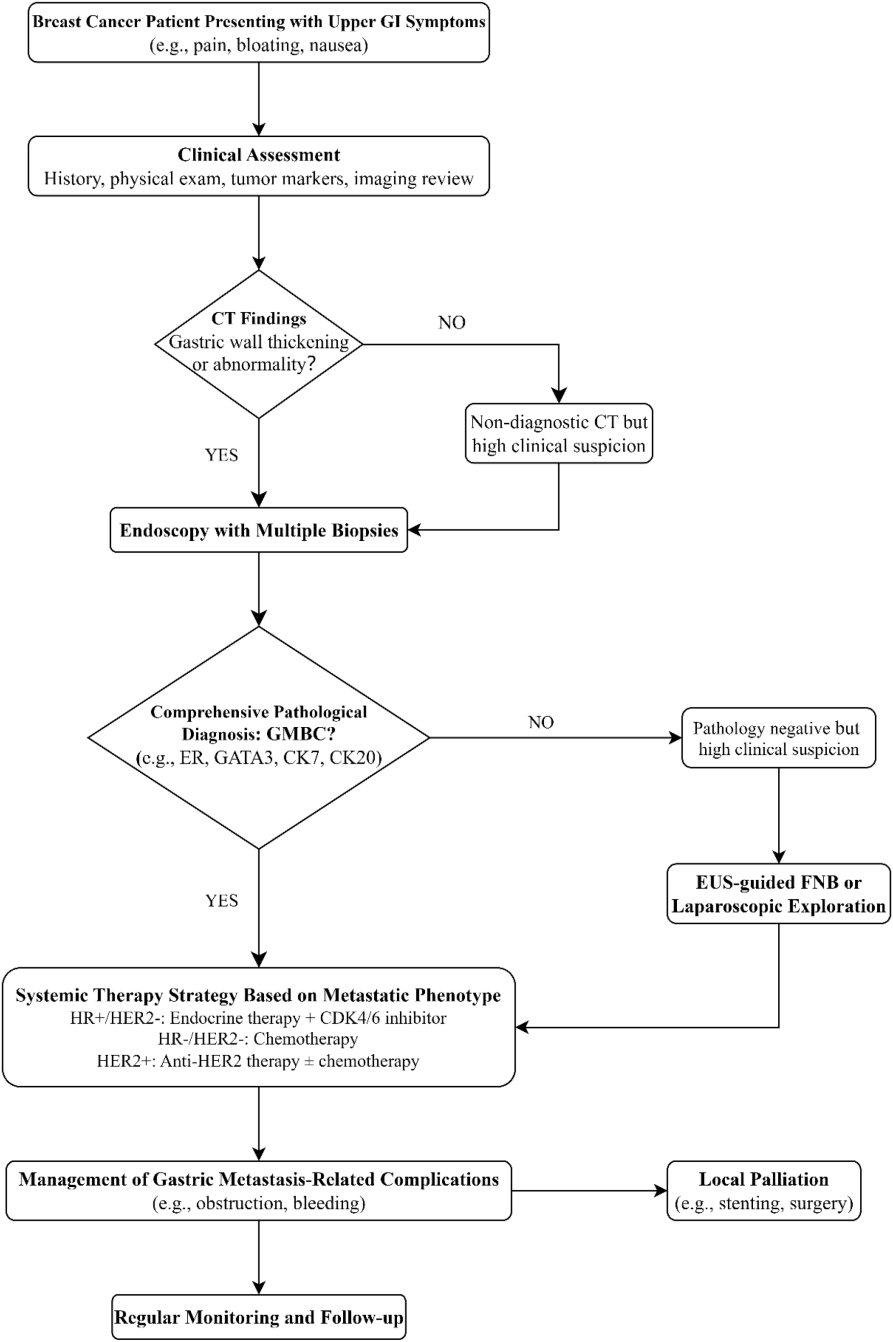
Proposed diagnostic and therapeutic algorithm for suspected GMBC.

To counter these pitfalls, we propose a structured diagnostic algorithm (**Figure 2**) for any breast cancer patient, particularly those with ILC, who develop new or persistent upper GI symptoms:

①Initial assessment: Correlate symptoms with tumor marker trends (e.g., Ca15-3, Ca125) and review recent CT imaging for subtle gastric wall changes or effusions.

②Imaging findings:

a. If CT is unequivocal for gastric pathology, proceed to gastroscopy with deep and multiple biopsies.

b. If CT is negative/non-diagnostic but clinical suspicion remains high (e.g., rising markers, persistent symptoms), proceed to gastroscopy.

③Pathological confirmation: Insist on a comprehensive IHC panel. A core panel should include ER, PR, HER2, GATA3, GCFDF15+, Mammaglobin,CK7, CK20, etc. (40–44) This combination is highly effective in distinguishing breast origin (e.g., GATA3+/ER+/CK7+/CK20-) from primary gastric cancer (e.g., typically GATA3-/ER-/CK7+/CK20+) (45).

④Handling discordant results: If clinical/radiological suspicion persists despite a negative superficial biopsy, pursue endoscopic ultrasound (EUS)-guided fine-needle biopsy to sample the submucosa and muscularis propria, where tumor cells often reside (5, 22).

### 4.2 The evolving tumor: implications of phenotypic discordance

A pivotal finding in our case was the change in the hormone receptor profile between the primary tumor (ER 80%, PR 5%) and the gastric metastasis (ER 30%, PR 0%). This phenomenon, observed in several cases from our review, underscores the concept of clonal evolution under therapeutic pressure. Treatment selectively eradicates therapy-sensitive clones, allowing resistant, potentially less-differentiated clones to proliferate at metastatic sites.

This finding mandates a paradigm shift in management. Re-biopsy of accessible metastatic sites at the time of progression is not optional but essential. It provides real-time biological data that can explain treatment resistance and guide subsequent therapy. A patient whose metastasis has lost ER expression is unlikely to benefit from further endocrine manipulation alone and may require a switch to chemotherapy.

### 4.3 Optimizing the therapeutic strategy

GMBC is a systemic disease, and management should be guided by the molecular subtype of the metastasis and the overall disease burden (**Figure 2**).

①Systemic therapy first: For most patients, especially those with multi-organ involvement, systemic therapy is the mainstay. For HR+ disease, endocrine therapy combined with CDK4/6 inhibitors remains the preferred option if the metastasis remains HR-positive (46, 47). For confirmed endocrine-resistant disease, chemotherapy (e.g., Eribulin, Capecitabine) is indicated.

②The role of local therapy: Surgery is generally reserved for palliation of complications like obstruction, bleeding, or perforation (37). However, for the highly selected patient with oligometastatic disease—where the stomach is the sole or dominant site of progression and the primary is controlled—curative-intent gastrectomy may be considered and has been associated with extended survival in some series (3). Endoscopic stenting is a less invasive alternative for managing gastric outlet obstruction (10).

## 5 Conclusion

GMBC is a diagnostic chameleon that demands vigilance. Our case and review highlight that overcoming this challenge requires a proactive and structured diagnostic approach, centered on endoscopy and definitive IHC profiling. Furthermore, the potential for phenotypic evolution between primary and metastatic sites makes re-biopsy a critical component of personalized cancer care, directly influencing therapeutic decisions. By integrating these principles—high clinical suspicion, rigorous pathological confirmation, and treatment tailored to the metastatic phenotype—clinicians can improve diagnostic accuracy and optimize outcomes for patients facing this complex manifestation of advanced breast cancer.

## Data Availability

The raw data supporting the conclusions of this article will be made available by the authors, without undue reservation.
